# Professional stakeholder’s views of adolescent weight management programmes: a qualitative study

**DOI:** 10.1186/s13104-021-05512-z

**Published:** 2021-04-03

**Authors:** Helen M. Jones, Oyinlola Oyebode, G. J. Melendez-Torres, Lena Al-Khudairy

**Affiliations:** 1 Warwick Medical School, University of Warwick, Coventry, CV4 7AL UK; 2 Peninsula Technology Assessment Group, College of Medicine and Health, University of Exeter, Exeter, UK

**Keywords:** Obesity, Adolescent, Weight management programme, Inductive content analysis, Stakeholder, Views, Qualitative, Engagement

## Abstract

**Objective:**

Family-based multi-component weight management programmes are recommended for adolescents with obesity in England and Wales, however, these programmes suffer from poor uptake and high attrition rates. This study aimed to gather the views of professional stakeholders in a UK weight management programme to identify potential areas to target to improve engagement and success for such programmes.

**Results:**

Semi-structured interviews were conducted with those involved in the commissioning, referral, coordination or delivery of a weight management programme (n = 11). Interviews were analysed using qualitative content analysis. Three main categories developed: professional support, tailoring and intervention content. Participants recognised the importance of support from experienced professionals, as well as family and peers. There was agreement that longer-term support was needed for adolescents with obesity; suggestions included integrating follow-up support with schools and leisure services. Emotional and psychological support must be prioritised. Having a variety of delivery modes, such as group and one to one, particularly in the home environment, were recommended. Stakeholders agreed that weight management programmes for adolescents need to be more proactive at incorporating technology. By acting on the views of those that work closely with adolescents, engagement with weight management programmes may be improved.

**Supplementary Information:**

The online version contains supplementary material available at 10.1186/s13104-021-05512-z.

## Introduction

Obesity is a well documented public health issue. 28% of children (2–15 years) in England are overweight or obese [1]. Not only are adolescents with obesity at greater risk of cardiovascular risk factors [2, 3], but psychosocial effects such as low self-esteem are evident [4]. Adolescents with obesity are likely to continue into adulthood with obesity [5]. Family-based multi-component weight management programmes are recommended for adolescents with obesity in England and Wales [6–9] with increased participation associated with increased weight loss [10]. However, these programmes suffer from poor uptake and high attrition rates [11–13]. Alongside adolescent views of weight management programmes, listening to those that work closely with adolescents with obesity on weight management programmes can give insight that might improve their future planning and delivery, in turn improving attrition and recruitment. This has been undertaken for other age groups previously [14–16]. However, the evidence base in terms of these stakeholders and their views of weight management programmes for adolescents, in the UK, is limited. This study aimed to gather the views of professional stakeholders in a UK weight management programme on potential areas to target to improve engagement and success of similar programmes, with specific reference to the delivery and content of Hearty Lives, a home-based weight management programme for families run by Wolverhampton City Council and part-funded by the British Heart Foundation. The practical implications of findings from a qualitative systematic review looking into the views of overweight and obese adolescents attending lifestyle obesity treatment interventions were also examined [17].

## Main text

### Methods

#### Data collection

Semi-structured interviews were conducted with those involved with the Hearty Lives programme from May to August 2018. Purposive sampling was used to obtain perspectives of information-rich participants relevant to the setting. Interviews took place by telephone or face-to-face in a private room at the participants place of work. All interviews were completed by one trained female researcher, undertaking a PhD, who had significant experience in weight management programmes and previous experience with qualitative methods (HMJ). Due to this prior experience, the researcher was known to some participants involved; this may have been advantageous in developing rapport and receiving honest views from stakeholders. Written consent was gained either electronically or in person. The interview guide was informed partly by a qualitative systematic review looking at the views of adolescents with overweight and obesity attending obesity treatment interventions [17]. Themes from this review were used to create a semi-structured interview guide, with an open mind for new themes emerging in the interviews. The interview guide was checked with two authors (GJM-T, LA-K), piloted prior to data collection and sent to stakeholders before the interviews commenced. Interviews were continued until data saturation was reached. A copy of the interview guide can be seen in Additional file 1.

#### Analysis

Data were transcribed verbatim and exported into NVivo 11 software anonymously. Data were approached using inductive qualitative content analysis [18]. Following open coding, codes were grouped together to form sub-categories, which were abstracted into categories. Categories were then named using words that closely linked to the data. Analysis was audited by another author (OO) to improve reliability. A draft version of the results was provided to stakeholders to allow member checking.

### Results

Interviews are described in Table 1.

**Table 1:** Participant characteristics and interview methods

Participant role	Number of participants	Number of participants attempted	Method of collection Interviews were on average 52 min (36–74 min)
Hearty Lives programme workers	N = 2	N = 2	Telephone interview (1) Face-to-face (1)
School nurses	N = 3	N = 13 8 did not respond, 2 had retired or left the trust	Face-to-face (2) Telephone interview (1)
Dietitians	N = 2	N = 2	Telephone interview (2)
Hearty Lives programme manager	N = 1	N = 1	Telephone interview (1)
Public health consultant	N = 1	N = 1	Telephone interview (1)
PE^a^ and school partner manager	N = 1	N = 1	Telephone interview (1)
Health advisor	N = 1	N = 1	Telephone interview (1)

^a^Physical Education

**Fig. 1:**
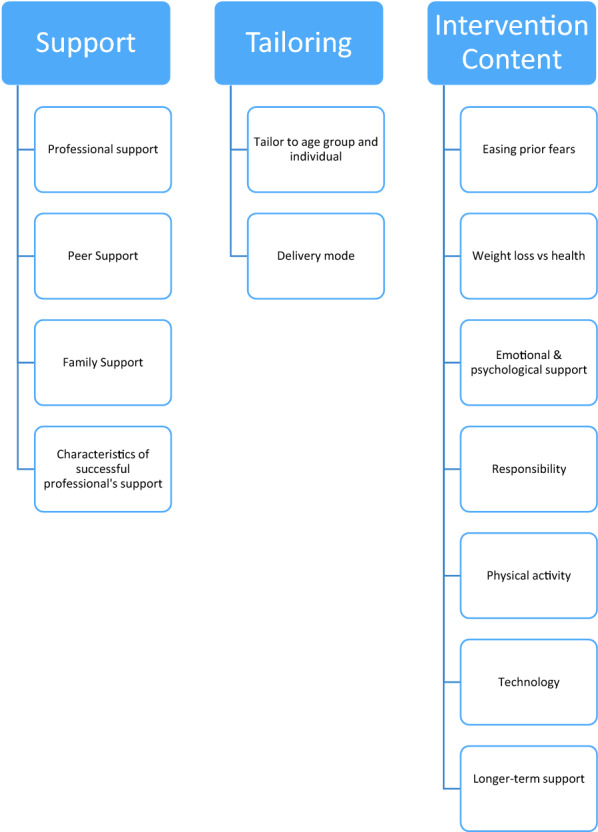
Categories and sub-categories

Analysis led to the development of three categories: professional support, tailoring and intervention content. These relate to what professional stakeholders consider important for future weight management programme design and delivery. Sub-categories within these categories are highlighted in bold throughout the text. All categories and sub-categories can be seen in Fig. 1. Transcribed quotations can be seen in Table 2.

**Table 2:** Representative quotes for sub-categories

Sub-categories	Representative quote
Category: support	
Professional support	*‘And I do believe that we need some, to have a good skill mix and, and multi skilled individuals.’* (S4)
Characteristics of successful professional’s support	*‘Yeah, I think there’s so much judgement around being overweight in the health professional world, you know? ‘Cause not all dietitians' are non-judgemental about overweight children. And when you get a parent who is so defensive and being so full of barriers my judgement of that parent is horrendous. So I think it is a very skilled area to work in because you have got to be non-judgemental at all times, even when you’re told ridiculous things.’* (S8)
Peer support	*‘People realise that they’re not the only ones who are struggling. And you can build friendship groups and create almost a, a cooperative around healthy lifestyle factors.’ (S4)*
Family support	*‘If the parent is open and willing to change, then the, then the teenager is. If the parent is defensive and, “You haven’t, your diet hasn’t worked for me,” or, “The GP doesn’t help,” or, “We’re all overweight.” Then the child, the teenager is very unreceptive.’ (S8)*
Category: tailoring	
Tailored to age group and individual	*But, yeah. It’s, it’s very hard and you do have to take a lot more time and consider the individual as well. 'Cause they are young adults at the end of the day. So it’s not, it’s not as simple as just going and, and picking something up last minute for the store cupboard and going and thinking, “Oh, that’ll be a good incentive.” You’ve got to try and think about the person that you’re working with (S2)* *‘I don’t think they particularly think about long term what it’s gonna do to their health. I think that it’s pretty much in the, it’s in the here and now isn’t it …how they feel at that time?’ (S9)*
Delivery mode	*‘I think, I think a mixture, because I think you would, you will get some, some young people that, especially if they’ve got, like, they’re really self-conscious, they wouldn’t want to be in a group situation. But then I think you’ve got others that would thrive in a group situation, because, you know, they know it’s not just them that’s, that’s, that’s, you know, maybe got a weight issue, or …I think it’s a little bit of, you know, when you’re with other people you can, you know, you can spur each other on, can’t you? Give each other encouragement and …I, I, I think a mixture.’ (S9)*
Category: intervention content	
Easing prior fears	*‘You, you could have a promotional site for the program where there might be clips on there of, video clips of what participants have done before and what people have said, participants have said. So, you know, I think people always do want information. Where would they go for information? I would imagine a website.’ (S1)*
Weight loss vs Health	*‘I think it doesn’t really matter what the primary motivation is, once you’ve got them there you can sell the message of how losing weight does help to improve their, their, their health. I think adolescents, as well as some adults, are concerned about their appearance, so if their appearance is driving them into your (coughs) excuse me, into their, into your arena, once you’ve got them there, it’s selling the health message.’ (S4)*
Emotional and psychological support	*‘I think that just shows that their self-esteem is so fragile that that needs to be the number one priority in the program is to build, build the self-esteem of the teenager.’ (S8)*
Responsibility	*‘Well, definitely at, at school, and in terms of what they eat between meals and things, I think they have sole control over it. Over, over the meals themselves the parents are the ones that buy the food. Yeah. So, so I think they, they, it’s going to be shared responsibility between the … it can't be all one, or all the other.’ (S10)*
Physical activity	*‘It’s about being free, it’s about easy access, it’s about being able to get there.’ (S5)*
Technology	*‘They love apps, anything like that, social media, you’ll, you’ll get them, you’ll grab them.’ (S5)*
Longer term support	*‘The groups need to go on for a long time for far more than a year to, to sustain real change I would think.’ (S8)*

#### Support

Professional stakeholders commented on the importance of qualified and experienced weight management professionals with a mix of skills including physical activity, nutrition and psychology. Stakeholders report that experienced staff instilled confidence in adolescents and parents. Stakeholders mention adolescents engaged better with ‘cool’ and relatable professionals. Non-judgemental characteristics were important with adolescents; stakeholders recognised that adolescents’ value being treated like an adult, which improve rapport, trust and engagement.

The consensus among stakeholders was that adolescents valued the peer support that came with attending a group programmes, leading to new bonds and friendships. Stakeholders report that exercising with others of a similar size made adolescents feel more comfortable.

It was felt that without family support, behavioural changes would be limited and educating the whole family facilitated longer sustainable lifestyle changes. Stakeholders report that adolescent’s personal responsibility and motivation increased if parents were engaged.

#### Tailoring

Stakeholders highlighted the importance of staff having the skills to be flexible and individually tailor sessions, recognising that adolescents need different support to younger children. The delivery mode of weight management programmes was spoken about in depth by all stakeholders. Many praised the home setting of the Hearty Lives programme because of its ability to tailor to each family, and to reach families who might not otherwise engage. However, although the benefits of 1–2–1 programmes were recognised, particularly in a home setting, several stakeholders felt that this option was not sustainable or time-efficient. Groups on the other hand offered a more cost-effective approach and were a good way to increase adolescents’ self-esteem. Both dietitians interviewed felt 1–2–1 programmes in clinic settings were not effective, mainly because of infrequent appointments, and formal atmosphere. Nonetheless, most stakeholders felt that the best option was to offer both group and 1–2-1 options accommodating the differing needs of adolescents and families.

#### Intervention content

Stakeholders reported that adolescents felt embarrassed and lacked confidence prior to attending weight management programmes. Suggestions to ease these prior fears included a taster session. Stakeholders suggested a promotional video for potential participants to watch and/or an induction session.

Stakeholders spoke about the importance of focusing on health within a weight management programme, even if weight loss was the adolescent’s motivation for initiating attendance.

One of the most well supported sub-categories within this analysis was the importance of ensuring all weight management programmes include support around emotional wellbeing, confidence and self-esteem. Stakeholders commented on how fragile adolescents were in terms of their self-esteem and confidence. With adolescence being a period of life with changing emotions and potential pressures, stakeholders suggested mental health support must be prioritised. Stakeholders felt that staff involved in weight management programmes and those who knew the adolescent well, should support their emotional well-being, rather than referring them on to external organisations. However, stakeholders were not comfortable speaking to adolescents about mental health without specific training.

The consensus amongst stakeholders was that adolescents need to take responsibility for their health and weight, but this should be shared responsibility with parents. Some stakeholders felt that adolescents do not realise it is their responsibility so incorporating this learning into an intervention could be promising.

Stakeholders’ general impression was that adolescents with obesity enjoy taking part in physical activity when completed as part of a weight management programme. This was felt to be a significant aspect of any weight management programme, kick starting engagement with physical activity. Stakeholders spoke of the importance of physical activity being fun. Helping adolescents to recognise that options for physical activity can include more than sessions in the gym. Activity must be tailored to the individual and not begin too strenuously. It should be affordable, free or subsidised. Accessibility was also noted as an important factor to encourage attendance. Stakeholders spoke about how adolescents feel in terms of poor confidence and body image, which leads to embarrassment when taking part in physical activity, particularly swimming. One criticism of the Hearty Lives programme was that it did not directly provide physical activity. Gender specific sessions were felt to be a good option if resources were available and several stakeholders commented on the competitive nature of some adolescents. A healthy level of competition, whether this was with family or friends, was motivating, reiterating the need for peer and family support.

All stakeholders spoke about incorporating technology into a weight management programme. Stakeholders agreed that online support should be part of a weight management programme, paired with face-to-face support. Stakeholders commented on the use of social media platforms as a way of supporting and promoting weight management programmes. Websites were also recommended but stakeholders commented on the need for commitment from staff to keep these up-to-date and continue promoting them. It seems stakeholders felt the idea of using technology, in principle, was something they should do, but were not currently doing well. The negative impact of social media in terms of poor body image was noted.

Most professionals commented on the need for longer-term support for adolescents. Hearty Lives was a 6-week programme with 12 months follow-up support at 3 monthly intervals; however, the initial programme and follow-up period was felt to be too short to ensure new habits were solidified. Stakeholders commented on integrating long-term support into other services. Examples included free gym or leisure centre passes for completers, or linking more with schools, including after school activity clubs.

### Discussion

In the current study, key categories were identified, which should be considered in the future when developing weight management programmes for adolescents with overweight or obesity. Professional stakeholders recognised the importance of support from experienced professionals, family and peers, when developing and delivering a tailored weight management programme for adolescents. This corresponds with findings from other qualitative studies of stakeholder’s perspectives towards child obesity treatment [14]. There was agreement amongst professional stakeholders that longer-term support was needed for adolescents with obesity, but also recognition of the restraints on resources to enable this. Insufficient resources and funding have also been reported with both pre-school and adult weight management services [15, 16]. Stakeholders felt that face-to-face support was still necessary, but suggestions included integrating follow-up support into schools and leisure services. This transition has also been suggested by stakeholders in Australia [19].

Additionally, the importance of easing prior worries about a programme is important to engage adolescents in the first place. The importance of professionals being non-judgmental was reported in this study and has been highlighted previously in a Canadian study [20]. Additionally, the need to offer adolescents emotional and psychological support within a weight management programme, in addition to nutrition and physical activity education was noted. This concurs with a recent systematic review investigating the views of adolescents attending obesity treatment interventions [17].

There was consensus amongst stakeholders that community-based weight management programmes worked best, as opposed to clinical settings. Having a variety of delivery modes, such as group and 1-2-1, particularly in the home environment, were recommended. Not only was the home setting of Hearty Lives praised in this study, other home-based weight management programmes have received positive feedback from adolescents [21]. The existing evidence suggests that there is no difference between individual or group-based programmes in terms of their effectiveness [7], however, a combination approach may warrant further investigation.

Stakeholders agreed that weight management programmes for adolescents need to be more proactive at incorporating an element of technology. This is encouraging, as text-messaging and web-based programmes have been positively reported previously [22–24].

Effective weight management programmes to support adolescents towards a healthy weight suffer from poor engagement. Studies exploring professional stakeholder views of adolescent weight management programmes in the UK are limited and tend to focus on younger children [14]. This study has highlighted key intervention components that are felt necessary by professional stakeholders.

#### Recommendations for practice and research

In order to improve engagement in adolescent weight management programmes, a family-based group programme delivered by experienced professionals may be warranted. Steps need to be made to ensure adolescents feel comfortable before attending a programme and programmes should be tailored to the adolescent age group and not include younger children. Emotional and psychological support should be incorporated into programmes that are carried out away from a clinical setting.

## Limitations

This study involved a small purposive sample of participants linked to a specific weight management programme in the West Midlands. This means the scope of their experience and knowledge may not include the full scope of what might be feasible nationwide or internationally.

## Supplementary Information

**Additional file 1. **Semi-structured interview guide. List of questions that were used as a guide to interview participants.

## Data Availability

The datasets generated and/or analysed during the current study are not publicly available due to the conditions of our informed consent but are available from the corresponding author on reasonable request within the next 10 years.
