# Supplementary material for: Professional stakeholder’s views of adolescent weight management programmes: a qualitative study
Source: BMC Res Notes. 2021 Apr 3;14:125. doi: 10.1186/s13104-021-05512-z (PMC8019507; doi:10.1186/s13104-021-05512-z)
Supplement: Supplementary file 1 — Additional file 1. Semi-structured interview guide. List of questions that were used as a guide to interview participants. [file 13104_2021_5512_MOESM1_ESM.pdf]

Understanding how best to support adolescents to achieve a healthy weight.

Interview guide

Prompt questions are in italics.

Start recording:

Hello. Thank you for taking part in this interview and for giving your time.

* The interview will take about 60 minutes.

* You can stop this interview at any time.

* I will record the interview.

* Your feedback will be used to develop programmes like Hearty Lives in the future, however your responses will be anonymous. It will not be possible to identify individuals from this interview.

* Are you happy to take part in the interview?

Questions for all interviewees related to Wolverhampton services e.g. Hearty Lives: **I am going to ask you some questions about weight management services for adolescents, specifically those targeting overweight or obese adolescents between 12 -17 years of age. Some questions relate specifically to the Hearty Lives weight management programme in Wolverhampton.**

1. How did you first hear about Hearty Lives?
2. What was your involvement with the Hearty Lives programme?
3. What are your general impressions or thoughts of the Hearty Lives programme?
4. What feedback did you receive from adolescents who took part in Hearty Lives/what feedback did you hear about the Hearty Lives programme if you didn't receive it directly from adolescents?
5. Is there anything that you think programmes like Hearty Lives could do differently/better?
6. Are you aware of any adolescent weight management services in surrounding areas?
    - *If yes, what are your thoughts of these services?*
7. What do you think about the current state of adolescent weight management services in Wolverhampton?
8. More research is needed to gain data from withdrawers, those that drop out from weight management programmes? How do you think this could be implemented?
9. Another issue is engaging overweight adolescents in the first place. What do you think stops overweight adolescents engaging in weight management services?
10. What are your thoughts regarding using online/technology support when working with overweight adolescents?
    - Text message/telephone/online forum/email support?

Questions for all interviewees related to systematic review:

As part of this research project, I have completed a systematic review that includes studies, which gathered the views of adolescents who have attended weight management interventions. All these adolescent views, which have come from 24 different weight management interventions across the world, have been combined. The next set of questions relate to findings from this systematic review:

1. The results of the SR highlighted importance of a weight management programme being tailored to a specific age group. Adolescents did not like attending weight management programmes when there were other children outside their age range (e.g. a programme for 8-16-year olds). What are your thoughts on this?
   - *How easy or difficult would this be to implement in practice?*
   - *Is there a need for them to be tailored to the adolescent age group?*
2. This SR showed that as well as adolescents wanting a programme that was tailored to their age group, tailored individual advice was important. Adolescents also valued having peer and group support. What are your thoughts on this?
3. The results of the SR highlighted the importance of physical activity and how much overweight and obese adolescents enjoyed taking part in physical activity within weight management programmes. What are your thoughts on this?
   - *Hearty Lives although, multi-component was more focused on behaviour change and diet. What are the practicalities of incorporating more physical activity into a weight management intervention?*
   - *Would this information from the systematic review make physical activity more of a priority when developing future programmes?*
   - *SR also highlighted cost was a big factor for adolescents engaging with physical activity. low cost or free gym access for example was a big draw to adolescents– what are your thoughts on this?*
4. A systematic review highlighted that many adolescents take part in weight management programmes because their family or professionals (e.g. school nurses, GP) have made them aware about the availability of a weight management intervention. What can be done to improve adolescents' awareness of weight management interventions?

5. The SR also showed that adolescents appreciated professionals that specialised in child weight management. This made adolescents feel like they were not the only ones with a weight issue and increased trust between adolescents and professionals. What are your thoughts regarding this?
   - *Is it always possible to have staff specialising in weight management?*
6. SR highlighted that some adolescents have prior fears of attending an intervention. Their expectations can be different to actual experience. For example, some felt that the physical activity element of a weight management programme would be very intense and military in style, perhaps from watching weight loss programmes on TV. What could be done to reduce this prior worry?
   - *As someone referring in to the HL programme, were you fully aware of the details of the programme and felt confident explaining this to the adolescent and their family?*
7. SR highlighted the desire for professionals to give more than just weight support, but support around self-esteem and building confidence. They valued having someone they could talk to about issues in addition to weight. How practical is this to implement?
   - *Prompt for those working with adolescents: How would you feel delivering this type of support*
8. Adolescents noted their primary motivation for attending weight loss interventions was weight loss rather than health. What do you think of this?
   - *The main driving force behind weight loss as the primary aim was mostly to do with adolescents wanting to improve their appearance. They felt this was the route to having more friends and reducing bullying. They felt that weight loss would lead to what they described as a more 'normal' life. What are your thoughts on this?*
9. Female adolescents have reported embarrassment of wearing sportswear and exercising in front of boys.
   - For those working directly: What is your experience of this?
   - For those working directly and those commissioning/managing: How practical could it be to have gender separate activity sessions?

ADDITIONAL FILE 1

Those referring in to Hearty Lives

These next questions are for those professionals who have had direct experience of referring adolescents into the Hearty Lives programme or similar programmes (if no, go to pink section below):

- How did you find the process of referring adolescents into the Hearty lives programme?
    - *How easy was this process? Any barriers to referring families into the programme?*
    - *Please describe your experience of engaging with adolescents and their families?*
- How comfortable were you with informing parents and adolescents about the hearty Lives programme?
    - *Did you have enough information?*
    - *Were posters, leaflets etc used?*
- Could anything be done to improve the referral system?
    - *Would you have benefited from more information?*

These questions are for those that do not have direct experience of referring into the Hearty Lives or similar programmes:

- What are your thoughts on the referral system for Hearty Lives or weight management programmes in general?
    - Could anything be improved?
    - What worked well?

To finish:

- Do you have any other comments about adolescent weight management interventions or anything else you would like to add?

ADDITIONAL FILE 1

Thank you for taking part in the study. Your contribution has been invaluable and we will be back in contact with details of any publications or papers that come out of this research.

NHS
National Institute for Health Research

3
Prevention and detection of diseases
